# Effects of Losartan on Renal Dopaminergic and Renin–Angiotensin System Alterations Induced by a High-Fat Diet in Sprague-Dawley Rats

**DOI:** 10.3390/ijms27209098

**Published:** 2026-10-14

**Authors:** Silvana M. Cantú, Hyun J. Lee, Christian Höcht, Adriana S. Donoso, Rodolfo Valtuille, Ana M. Puyó, Marcelo R. Choi

**Affiliations:** 1 Universidad de Buenos Aires, Facultad de Farmacia y Bioquímica, Departamento de Ciencias Biológicas, Cátedra de Anatomía e Histología, Buenos Aires 1113, Argentinamchoi@ffyb.uba.ar (M.R.C.); 2 Universidad de Buenos Aires, Facultad de Farmacia y Bioquímica, Instituto de Fisiopatología y Bioquímica Clínica (INFIBIOC), Laboratorio de Síndrome Metabólico Experimental, Buenos Aires 1113, Argentina; 3 Universidad de Buenos Aires, Facultad de Farmacia y Bioquímica, Departamento de Farmacología, Cátedra de Farmacología, Buenos Aires 1113, Argentina; 4 Pontificia Universidad Católica Argentina, Especialización en Nefrología, Facultad de Ciencias Médicas, Buenos Aires 1113, Argentina; rvaltuille@gmail.com; 5 Universidad de Buenos Aires/Consejo Nacional de Investigaciones Científicas y Técnicas (CONICET), Instituto Alberto C. Taquini de Investigaciones en Medicina Traslacional (IATIMET), Buenos Aires 1113, Argentina

**Keywords:** renal dopaminergic system, high-fat diet, blood pressure, inflammation, losartan, rats

## Abstract

The renal dopaminergic system (RDS) promotes natriuresis, diuresis, and exerts anti-inflammatory effects. High-fat diet (HFD) intake triggers the renin–angiotensin system (RAS). The aim of this study was to determine whether losartan (L) prevents RDS-RAS alteration and its impact on blood pressure and renal injury induced by HFD. Male Sprague–Dawley rats studied for 8 weeks were assigned to four groups: control (C; n = 4); HFD (n = 6): 50% *w*/*w* of bovine fat added to the standard diet; control + losartan (CL; n = 4): 30 mg/kg/day of L dissolved in drinking water; and HFD + losartan (HFDL; n = 6): HFD + CL. (Mean ± SD; *p* < 0.05). L prevented the following: HFD-induced increases in systolic blood pressure (SBP) and L-Dopa/dopamine urinary index (HFD vs. C, *p* < 0.01; HFDL vs. HFD, *p* < 0.01); lower expression of OCTN-1/2/3 (HFD vs. C, *p* < 0.01; HFDL vs. HFD, *p* < 0.05); overexpression of D1DR and Na^+^/K^+^-ATPase (HFD vs. C, *p* < 0.01; HFDL vs. HFD: D1DR *p* < 0.01; Na^+^/K^+^-ATPase *p* < 0.05), NF-κB1 and TGF-β1 (HFDL vs. HFD, *p* < 0.01). L mitigated proximal tubular alterations and interstitial fibrosis (HFDL vs. HFD, *p* < 0.01). Under chronic HFD intake, early L administration prevented renal dopaminergic system alterations and SBP elevation, and attenuated renal inflammation and interstitial fibrosis, reflecting its nephroprotective effects.

## 1. Introduction

Worldwide, saturated fatty acids account for approximately 10% of total daily energy intake, with values ranging from 2.3% to 27.5% across countries, reaching the highest levels in upper-middle- and high-income countries, where ultra-processed foods predominate in dietary patterns [1]. Such diets are strongly associated with an increased risk of cardio-reno-metabolic disorders, including coronary heart disease, endothelial dysfunction, insulin resistance, and progressive renal injury, largely mediated by their pro-inflammatory, dyslipidemic, and oxidative effects [2]. Consequently, the World Health Organization (WHO) recommends limiting saturated fat intake to less than 10% of total energy intake to mitigate the risk of cardiovascular and metabolic diseases [3].

Accordingly, the consumption of a high-fat diet (HFD) is one of the major factors contributing to the development of metabolic syndrome (MS). This clinical entity is defined by the presence of at least three of the following metabolic abnormalities: hyperglycemia, hypertriglyceridemia, insulin resistance, elevated blood pressure, increased waist circumference, and systemic inflammation, which affects multiple organs, including the kidneys, which are essential for hydro-electrolytic homeostasis and blood pressure regulation, among other functions [4,5]. HFD intake induces structural and functional renal alterations and contributes to the development of arterial hypertension, a chronic and often asymptomatic disease that accounts for almost 50% of global cardiovascular mortality [6,7].

Renal sodium handling plays a critical role in blood pressure regulation, a process controlled by several neurogenic, autocrine and endocrine factors, which can be divided into two opposing categories: those promoting vasoconstriction and sodium retention, and those driving vasodilation and natriuresis [8,9]. Traditionally, hyperactivation of the intrarenal RAS, through AT1 receptors (AT1R), has been recognized as a primary driver of sodium reabsorption, renal vasoconstriction, and hypertension development [10]. In contrast, several studies underline the protective role of the renal dopaminergic system (RDS) as a counter-regulator to the hypertensive actions of angiotensin II (Ang II) [9]. The RDS, located primarily in the proximal convoluted tubules (PCT), promotes natriuresis and diuresis through D1-like (D1DR) receptors (D1, D3, and D5), and exerts anti-inflammatory effects through D2-like (D2DR) receptors (D2 and D4), thereby counteracting the well-known antinatriuretic and pro-inflammatory effects of Ang II [11]. Therefore, these two systems exhibit functional antagonism, and one of the main targets of both systems is the renal pump Na^+^, K^+^-ATPase, an enzyme whose alteration has been associated with sodium retention and hypertension [12,13]. Considering that L-dopa, the precursor of dopamine, and dopamine itself are excreted in the urine and both compounds can be quantified, the urinary L-dopa/dopamine ratio has been proposed as an early biomarker of renal damage in animal models of fructose-induced arterial hypertension [14].

Antihypertensive therapy includes several pharmacological treatments. Among them, losartan (L) is a safe and potent selective AT1R antagonist, well known for its high patient adherence and reliable clinical outcomes [15]. Although its antihypertensive efficacy is well established, whether losartan can modulate the RDS–RAS interaction in HFD-induced MS as part of its nephroprotective action remains unclear.

Considering all this evidence, we hypothesized that the development of arterial hypertension and renal injury in rats with an MS-like phenotype induced by an HFD may be associated with an impaired relationship between the RDS and the RAS, characterized by reduced endogenous intrarenal dopamine availability and enhanced RAS through AT1R, which promotes sodium retention and renal inflammation. Given the fact that Ang II inhibits renal dopamine activity [11], we further hypothesized that pharmacological blockade of the RAS with losartan would prevent all these renal alterations induced by HFD. We also assessed the potential utility of the urinary L-DOPA/dopamine ratio as a marker and/or predictor of renal dysfunction in states characterized by sodium retention, insulin resistance and/or hypertension.

## 2. Results

Table 1 shows that HFD significantly increased body weight vs. C (473.8 ± 39.1 vs. 415.7 ± 48.9 g; *p* < 0.05), an effect prevented by losartan in HFDL (359.1 ± 38.2 vs. 473.8 ± 39.1 g; *p* < 0.05). Losartan prevented the reduction in food intake in HFDL vs. HFD (18.6 ± 4.8 vs. 13.8 ± 2.2 g/24 h; *p* < 0.05), whereas caloric intake increased in HFD (103.6 ± 28.4 vs. C: 60.5 ± 26.9 kcal/24 h; *p* < 0.05) with differences between HFDL vs. HFD (119.3 ± 10.5 vs. 103.6 ± 28.4; *p* < 0.05). Water intake did not differ among groups. Plasma metabolic parameters increased significantly in HFD vs. C, with reductions in triglycerides and insulin in HFDL (83.0 ± 13.9 vs. 119.3 ± 9.6 mg/dL; *p* < 0.01; and 2.8 ± 0.1 vs. 4.2 ± 0.5 ng/mL respectively; *p* < 0.05). Also, triglycerides showed a reduction in the losartan groups CL vs. HFDL, implying that losartan can modify this parameter by itself (53.2 ± 10.2 vs. 83.0 ± 13.9; *p* < 0.05). Regarding glucose, it was increased in HFD vs. C (152.2 ± 12.3 vs. 117.5 ± 17.3; *p* < 0.05), and losartan reduced this metabolite in CL vs. C (100.3 ± 23.0 vs. 117.5 ± 17.3; *p* < 0.05) but not in HFDL vs. HFD. Furthermore, triglyceride–glucose index (TyG) and homeostatic model assessment for insulin resistance (HOMA-IR) were also significantly increased in HFD vs. C (10.5 ± 0.1 vs. 8.0 ± 0.4 and 3.3 ± 0.6 vs. 0.7 ± 0.1 respectively, *p* < 0.01), with no significant changes using losartan.

In Table 2, HFD increased SBP and the urinary L-Dopa/dopamine index vs. C (134.8 ± 7.6 vs. 120.7 ± 1.4 mmHg; *p* < 0.01; and 2.9 ± 0.8 vs. 1.1 ± 0.4; *p* < 0.05, respectively); both effects were attenuated by losartan in HFDL. CL exhibited a further SBP reduction vs. C. urinary albumin/creatinine ratio (UACR) and creatinine clearance (ClCr) did not differ among groups. Diuresis, sodium fractional excretion (FENa^+^), and daily urinary sodium excretion (UNa^+^.V) decreased in HFD (3.5 ± 0.7 vs. 16.4 ± 3.6 mL/24 h, 0.23 ± 0.01 vs. 0.43 ± 0.06% and 0.5 ± 0.2 vs. 1.8 ± 0.3 mEq/24 h; respectively *p* < 0.01) and those effects were prevented by losartan in HFDL.

Histological analysis with hematoxylin–eosin staining revealed cytoplasmic vacuoles in cortical tubular cells in HFD vs. C, with no changes in CL vs. C; HFDL displayed smaller vacuoles than HFD (Figure 1). Using transmission electron microscopy, podocyte ultrastructure showed foot processes shortening and fusion in HFD, prevented by losartan in HFDL, with no differences between CL and C (Figure 2). These microscopic and ultrastructural observations represent qualitative and descriptive findings of early focal alterations.

Protein expression of RDS by Western blot analysis revealed that L-amino acid transporter type 2 (LAT2) and organic cation transporter type 2 (OCT2) did not vary among groups. Also, HFD reduced novel organic cation transporters (OCTN1,2,3) vs. C, while losartan prevented this decrease. Losartan also prevented D1DR overexpression in HFDL vs. HFD. Regarding D2DR, losartan increased its expression in CL vs. HFDL (Table 3 and Figure 3).

Considering RAS, HFD overexpressed AT1R with no changes with losartan. Finally, Na^+^/K^+^-ATPase expression was increased in HFD, and losartan could prevent this in HFDL vs. HFD (Table 3 and Figure 3).

The L-Dopa/dopamine index correlated positively with SBP, insulin, and D1DR, and inversely with Una^+^.V and OCTN1,2,3. Losartan prevented these alterations in HFDL vs. HFD (Figure 4).

Regarding inflammatory markers, HFD significantly increased NF-κB1 and TGF-β1 expression, effects prevented by losartan in HFDL. Furthermore, losartan itself decreased TGF-β1 in CL vs. C (0.82 ± 0.47 vs. 4.43 ± 1.29; *p* < 0.01) (Table 4 and Figure 5).

In addition, interstitial fibrosis increased in HFD vs. C and was significantly reduced by losartan in HFDL vs. HFD, with residual *foci* and no changes in CL vs. C (Figure 6). Semi-quantitative analysis confirmed these findings (Figure 7). The observed results were C = 15.25 ± 1.92; HFD = 28.98 ^†^ ± 3.75; CL = 20.45 ^¶^ ± 3.21; HFDL = 20.37 ^§^ ± 3.09. (Data: Mean ± SD. Significance: ^†^ *p* < 0.01 C vs. HFD; ^§^ *p* < 0.01 HFDL vs. HFD; ^¶^ *p* < 0.01 CL vs. C).

## 3. Discussion

The combination of unhealthy habits, such as a sedentary lifestyle and consumption of an atherogenic diet, contributes to the development of hypertriglyceridemia, insulin resistance, elevated SBP, and systemic inflammation, among other metabolic alterations. It has been demonstrated that diets rich in saturated fats affect multiple organs, including the kidneys, which participate in several regulatory processes, including blood pressure control, through the balance between the RDS and the RAS. In addition, losartan, a widely used antihypertensive drug, has demonstrated high patient adherence and reliable therapeutic outcomes.

In this experimental model, a significant increase in body weight was observed in the HFD group compared with the C group, and this increase was effectively prevented by losartan. Although food intake was lower in the HFD group, the higher caloric density of the diet resulted in an approximately 33% higher caloric intake, thereby accounting for the observed body weight gain and being consistent with the findings of Pinhal et al. [16]. Our findings agree with those previously reported by Smith et al. [17], who observed a reduction in body weight following losartan administration in HFD-fed rats, suggesting a modulatory role of AT1 receptor antagonism in diet-induced obesity. Furthermore, Sharieh Hosseini et al. [18] proposed that losartan may improve leptin resistance and modulate body weight in diet-induced obesity. Water intake did not differ among the experimental groups, and, to date, there is no evidence that losartan affects this parameter. An important methodological consideration in our study is the potential confounding effect of body weight on the observed renal and metabolic outcomes. Given that losartan completely prevented HFD-induced body-weight gain despite a persistently high caloric intake, it should be considered that the structural and functional renal protection observed in the HFDL group could be secondary to a reduced adipose mass or an altered systemic metabolic state, rather than a direct intrarenal AT1R/RDS interaction. Nonetheless, our data reveal a clear dissociation between systemic metabolic derangements and local renal protection. Despite their lower body weight, HFDL rats exhibited fasting glucose, TyG index, and HOMA-IR values that were statistically indistinguishable from the HFD group, confirming that a systemic state of insulin resistance and metabolic dysfunction persisted in these animals. Moreover, losartan effects in the kidney were selective; it rescued OCTN1,2,3 expression and normalized D1DR and Na^+^/K^+^-ATPase levels without altering the HFD-induced overexpression of the AT1R receptor. This specific response underscores that while body weight is a notable covariate, the preservation of renal architecture, podocyte ultrastructure, and natriuretic capacity in our experimental model seems to be predominantly driven by direct AT1R blockade and the disruption of local AT1R-D1DR negative crosstalk within the kidney.

The metabolic profile indicated the presence of insulin resistance in the HFD-fed group, as evidenced by elevated triglyceride, glucose, and insulin levels, as well as increased TyG and HOMA-IR indices, in agreement with previous MS-like phenotype models induced by HFD administration [19]. On the other hand, losartan partially attenuated the increases in triglycerides and insulin, without modifying glucose levels or the TyG and HOMA-IR indices, whereas the CL group did not differ from the C group. A possible mechanism underlying the beneficial effects of losartan on the observed metabolic alterations may be related to a reduction in oxidative stress through SOD activation, although the exact mechanisms remain uncertain [17,20].

Regarding hemodynamic parameters, HFD intake significantly increased SBP, consistent with the findings of Jin et al. and Li et al. [21,22], who associated HFD consumption with RAS activation, increased Ang II and aldosterone levels, and upregulation of AT1R expression, together with renal inflammation and fibrosis. Concomitantly, losartan administration prevented the increase in SBP, consistent with the effects expected from AT1R antagonism [17]. Furthermore, losartan was able to reduce SBP in normotensive control rats, in agreement with Lee et al. [19]. Li et al. [22] also described an allosteric effect of losartan at AT1R that enhances D1DR activation, thereby contributing to its antihypertensive action.

Regarding renal filtration, estimated by ClCr, it remained within the normal range across all experimental groups. This observation is consistent with the findings of Roza et al. [23] in mice fed an HFD for 8–12 weeks, whereas other studies have reported hyperfiltration in obese animals [24,25]. Thus, the effects of HFD on ClCr may vary depending on the experimental model and conditions. In addition, our findings indicate that losartan did not modify ClCr in our model. However, we also recognize that since our model demonstrates a dysregulation of OCTN1,2,3 by HFD and its prevention by losartan, the baseline kinetics of tubular creatinine secretion could potentially fluctuate among the experimental groups, introducing a confounding factor that limits its interpretation as an estimator of the glomerular filtration rate [26].

The UACR, which is considered more reliable than isolated urinary albumin measurements [27], remained below 30 mg/g in all experimental groups. Although 30 mg/g is the established clinical threshold for moderately increased albuminuria in humans, its direct applicability to rodents has not been validated. However, the values observed in the present study did not exceed the human clinical threshold used as a reference, and losartan administration did not significantly alter this parameter. In contrast, Sánchez-Navarro et al. [28] reported increased UACR following longer HFD exposure, suggesting that the duration of exposure may be a determining factor in the development of albuminuria in this MS-like phenotype model.

This experimental model also showed decreased diuresis in the HFD-fed group, supporting previous observations by Sánchez-Navarro et al. [28], possibly because of an imbalance between RDS and RAS [14]. Losartan prevented this reduction, suggesting preservation of RDS alteration in association with AT1R blockade [22]. In addition, FENa^+^ and UNa^+^.V were decreased in the HFD group, and these reductions were partially prevented by losartan, suggesting that sodium reabsorption mechanisms independent of Na^+^/K^+^-ATPase may have remained active, such as the Na^+^/H^+^ exchanger or ENaC [29,30]. In this regard, previous findings are controversial: Roza et al. [23] observed no changes, whereas Pinhal et al. [16] reported decreased FENa^+^ and UNa^+^.V in obesity or following prolonged HFD intake.

Histological analysis showed that HFD induced the formation of cytoplasmic vacuoles in proximal tubule cells, which were absent in the C group, whereas losartan partially prevented this alteration, with no changes observed in the CL group. Although the lipid stains used (Sudan Black and Oil Red O) were negative, the literature suggests that lipid vacuoles [7,31] or hydropic degeneration may result from Na^+^/K^+^-ATPase dysfunction associated with lipotoxicity [32]. This alteration was attenuated by losartan, potentially through reductions in inflammation and oxidative stress [20]. Ultrastructural analysis by TEM revealed alterations in podocyte foot processes, including effacement and interdigitation, in response to HFD intake; these alterations were partially prevented by losartan, consistent with Ang II-mediated injury through AT1R [33] and with the reported protective role of renal dopamine [34,35].

The HFD-induced MS-like phenotype is associated with elevated blood pressure mediated by RAS activation and the action of Ang II on AT1R, which stimulates Na^+^/K^+^-ATPase and thereby favors sodium retention [4,23]. Concomitantly, HFD suppresses RDS activity, thereby increasing anti-natriuresis. A negative correlation exists between AT1R and D1DR in proximal tubular cells, whereby activation of one receptor induces internalization and loss of signaling of the other, causing them to function as opposing units with Na^+^/K^+^-ATPase as a common target [36]. In normotensive humans, the negative interaction between RDS and RAS in renal sodium transport depends on sodium intake [37]. However, HFD intake increased renal AT1R expression, consistent with MS models and RAS activation [22]. Losartan did not prevent AT1R overexpression in the HFDL group after 8 weeks, although a tendency toward a reduction was observed, suggesting that a longer treatment period may be necessary. In contrast, losartan prevented the increase in the urinary L-dopa/dopamine index observed in the HFD group [38], implying greater tubular dopamine availability and urinary excretion, associated with restoration of OCTN1,2,3 transporter expression. LAT2 and OCT2 transporter expression remained unchanged, in contrast to the effects observed under fructose overload conditions [38]. Regarding L-dopa transporters, we proceed to evaluate specifically the Na^+^-independent L-type amino acid transporter 2 (LAT2) given its relevance as the rate-limiting pathway for the uptake of circulating L-dopa in the proximal tubule cells [39]. Once L-dopa is intracellularly decarboxylated into dopamine, this monoamine acts as an organic cation at physiological pH. There are several polyspecific organic cation transporters (OCT 1, 2 and 3 basolaterally and OCTN 1,2,3 apically/luminal) that are key components in regulating the directional transit, secretion, and spatial availability of dopamine within the renal proximal tubule. We chose to evaluate OCT-2 and OCTN1,2,3 based on previous experiments performed in our laboratory using a similar experimental model based on fructose overload [38].

Although the present experimental design shares several features with our previous fructose-overload model [38], the metabolic insults underlying the two models differ. In fructose overload, renal fructose metabolism, particularly in the proximal tubule, can promote ATP depletion and uric acid generation, which have been associated with oxidative stress, activation of the intrarenal RAS, and altered renal sodium handling [38]. In contrast, high-fat feeding primarily increases the renal exposure to fatty acids and may promote ectopic lipid accumulation and lipotoxicity, with downstream mitochondrial dysfunction, oxidative stress, inflammation, and fibrotic remodeling [40,41,42]. Therefore, although both models may converge on common pathways such as RAS activation, oxidative stress, inflammation, and altered renal sodium handling, the upstream metabolic mechanisms are distinct.

HFD increased D1DR expression, an effect that was effectively prevented by losartan, while D2DR expression decreased and remained unaffected by losartan, reflecting the disruption of renal dopaminergic signaling induced by diet [43]. Regarding D1R and D2R receptors, the extensive literature on renal physiology confirms that while the kidney expresses all five dopamine receptor subtypes, the D1DR and D2DR are the most abundantly expressed and robustly active subtypes within the renal cortex, and are mainly responsible for mediating dopamine’s intrarenal actions, both hemodynamic/natriuretic (D1DR) and cellular/anti-inflammatory effects (D2DR) [44]. It has been described that D1DR accounts for almost all of the cAMP production since D1DR-specific gene silencing inhibits cAMP accumulation induced by activation of the D1-like receptor, whereas D5DR siRNA has no effect on the D1-like receptor-mediated increase in cAMP production. Moreover, D1R depletion, using D1R siRNA, blocks the D1-like receptor-dependent inhibition of sodium transport [45,46]. D2DR is the classic prototype for the D2-like family and is also implicated in dopamine-natriuretic effects, but its effect seems to be associated with its antioxidant and anti-inflammatory action. The D2DR-mediated regulation of oxidative stress involves both pro-oxidant and antioxidant systems, since it has been described that it increases the expression of antioxidant enzymes such as sestrin2 and decreases renal reactive oxygen species (ROS) production by reducing NADPH oxidase expression and activity [47,48].

Taken together, these findings suggest that losartan may attenuate HFD-associated alterations in urinary catecholamine handling and RDS-related protein expression.

Cortical Na^+^/K^+^-ATPase expression was increased by HFD [14], and this increase was prevented by losartan without altering baseline expression, consistent with the proposed allosteric interaction between AT1R and D1DR mediated by losartan [22].

The urinary L-dopa/dopamine index was positively associated with SBP and insulin and negatively associated with UNa+.V and OCTN1,2,3 across the complete experimental dataset, suggesting an association between urinary catecholamine handling and blood pressure, sodium balance, and insulin resistance [49]. In losartan-treated animals, these associations were no longer observed, in parallel with AT1R blockade and reduced Na^+^/K^+^-ATPase expression. Collectively, these associations should be interpreted as relationships observed across the experimental conditions and not as evidence of a direct relationship within each individual treatment group.

Regarding inflammation, the RDS exerts anti-inflammatory effects, whereas the RAS promotes inflammation [11,15]. Renal dopamine, through D1DR and D2DR signaling, can attenuate the effects of Ang II by reducing inflammation and fibrosis [15]. HFD induced a pro-inflammatory environment characterized by NF-κB1 and TGF-β1 overexpression, increased interstitial fibrosis, and activation of oxidative pathways [6,42,50]. Conversely, losartan reduced fibrosis and oxidative stress [51].

In summary, preventive and continuous administration of losartan was associated with attenuation of HFD-induced alterations in RAS- and RDS-related parameters. AT1R blockade by losartan was associated with prevention of the HFD-induced increase in Na^+^/K^+^-ATPase expression and maintenance of D1DR expression within the range observed in control animals, while OCTN1,2,3 expression was also preserved. These changes were accompanied by increased urinary dopamine excretion and prevention of the HFD-induced increase in the urinary L-Dopa/dopamine index, together with improved natriuretic parameters. Collectively, these findings are consistent with an improvement in renal dopaminergic handling and partial restoration of the RDS–RAS balance following losartan treatment. In addition, losartan ameliorated interstitial fibrosis and preserved proximal tubular structure and podocyte ultrastructure.

In conclusion, early administration of losartan in an HFD-induced MS-like phenotype was associated with attenuation and, for several parameters, prevention of the functional, structural, and ultrastructural renal alterations induced by HFD. These effects were accompanied by changes in RDS- and RAS-related parameters consistent with partial restoration of the balance between these two systems. Overall, these findings suggest a potential nephroprotective role of early losartan treatment in this experimental model.

### Study Limitations

We presented the results obtained after 8 weeks of treatment, which may be considered a relatively short experimental period for this type of study. This time point was selected based on previous findings obtained at two additional experimental time points, 4 and 12 weeks, using the same experimental design and methodology [14]. At 4 weeks, the metabolic phenotype under investigation had not yet fully developed, whereas by 8 weeks it was clearly established and persisted through 12 weeks. Therefore, 8 weeks was considered the earliest time point at which all the determinations included in the present study could be reliably performed, while ensuring that the metabolic phenotype of interest was fully established.

Another limitation of our study is the small sample size per group, particularly for the control (C) and losartan (CL) groups. We determined the number of animals based on previous experimental studies using the same model, while also adhering to the laboratory animal ethics committee’s strict requirements regarding the minimization of animal use.

Another limitation of the present study is that only male rats were included, based on our previous experience with this experimental model. We acknowledge, however, that the inclusion of female animals would have provided valuable complementary information regarding potential sex- and estrogen-dependent effects of both the diet and the pharmacological treatment. In addition, a more comprehensive functional assessment of the RDS would have strengthened the interpretation of our findings. In particular, direct assessment of dopamine synthesis and/or downstream signaling, as well as a more direct evaluation of AT1R signaling, would have provided further mechanistic insight into the alterations observed in the present study.

Given that the animals in each experimental group were housed in groups (in order to reduce stress due to chronic individual isolation), losartan was administered at a dose of 30 mg/kg/day in the drinking water (and not by gavage in order to minimize another source of stress) for all the animals placed in the same cage. This could represent a potential source of variability regarding the dose received by each animal.

Regarding diet composition, it is important to mention that the interpretation of the present findings is based on the overall metabolic, hemodynamic, renal, and structural phenotype induced by the dietary intervention, and we do not attribute the observed effects exclusively to dietary fat while excluding possible contributions from differences in other dietary components. This limitation does not imply that a specific amino acid or micronutrient deficiency may occur in our animals, as these parameters were not directly assessed. Rather, it indicates that a potential contribution of differences in nutrient intake cannot be completely excluded and should be considered when interpreting the effects of the HFD in this experimental model.

Regarding SBP measurement, tail-cuff plethysmography could be considered an inappropriate method for this protocol, but with all the precautions taken to perform it, the values obtained are reliable.

Another limitation of our study is the use of creatinine clearance (ClCr) as an indicator of renal filtration. This carries limitations, as it may slightly overestimate the true glomerular filtration rate due to a certain degree of active tubular creatinine secretion in rodents [52]. Additionally, renal filtration values across different experimental groups should be interpreted carefully when estimated from endogenous ClCr, since it reflects a comprehensive balance of both glomerular filtration and steady-state tubular transport under an 8-week protocol, in which baseline kinetics of tubular creatinine secretion could potentially fluctuate among the experimental groups due to dysregulation of OCTN1,2,3, introducing a potential confounding factor.

Regarding Na^+^/K^+^-ATPase, only its protein expression was assessed globally, without distinguishing between the phosphorylated active form and the non-phosphorylated inactive form of this protein. Another limitation of our study is that we did not determine Na^+^/K^+^-ATPase activity, indicating that changes in its protein expression are not necessarily reflected in changes in its enzymatic activity.

Direct assessments of oxidative stress could have been performed for the present study due to the known effect of HFD and the possibility that losartan reduces insulin through the reduction in oxidative stress; however, priority was given to evaluating inflammatory markers and fibrosis, as these parameters are directly linked to renal structural integrity and function, which were affected by HFD and ameliorated by losartan.

Although several aspects of this complex system remain to be elucidated and our study has inherent limitations, our findings contribute new information to a relatively overlooked area of research. We hope that these observations will help renew interest in this “old-new” field and stimulate further studies aimed at clarifying its physiological and pathophysiological relevance, with the longer-term prospect of exploring its potential translational and clinical implications.

## 4. Materials and Methods

### 4.1. Animals, Experimental Design, and Diet

Male Sprague–Dawley rats (6 weeks old, 180–200 g; Central Animal Facility, Facultad de Farmacia y Bioquímica—Universidad de Buenos Aires) were housed under controlled conditions (22 ± 2 °C; 50–70% humidity; 12 h light/dark) and studied for 8 weeks, randomized into four experimental groups (n = 4–6). Given that all the rats were of the same sex and age and had similar weights, each animal had an equal probability of being directly assigned to any experimental group.

Experimental groups:

- C: standard diet and water *ad libitum* (SD) (n = 4).
- HFD: SD + 50% *w*/*w* bovine fat and water *ad libitum* (n = 6).
- CL: C + losartan (L; 30 mg/kg/day, in drinking water) (n = 4).
- HFDL: HFD + L as above (n = 6).

Table 5 and Table 6 show detailed diet composition, and Table 7 shows detailed losartan information.

### 4.2. Body Weight, Intake, and Calories

Body weight was recorded daily. Food and fluid intake were measured every 48 h. Calculations:

(I) *food* (g/day) = *offered* − *leftover*;
(II) *fluid* (mL/day) = *offered* − *leftover*;
(III) *calories*: *C and CL* = *SD* (g) × 3.3 kcal/g;
(IV) *HFD and HFDL* = *SD* (g) × 3.3 kcal/g + *fat* (g) × 9 kcal/g.

### 4.3. Measurement of Weight and Fluid Intake and Estimation of Losartan Concentration

Considering the total body weight of the animals in each cage and the differences in the daily liquid intake between rats receiving a high-fat diet with respect to control rats, it was necessary to adjust the losartan concentration to give the same daily dose of losartan to all the experimental groups under treatment with this drug. To do this, the weight of the rats in group CL and group HFDL was determined daily, as well as their intake of drink, to adjust the concentration of losartan in the drinking water according to the following formula:

$$\mathrm{Losartan}\;\mathrm{weight}\;\left(\mathrm{mg}\right)=\frac{30\;\mathrm{mg}}{\mathrm{kg}/\mathrm{day}}\times \frac{\mathrm{body}\;\mathrm{weight}\;(\mathrm{kg})}{\mathrm{liquid}\;\mathrm{intake}/\mathrm{day}\;(\mathrm{mL}/\mathrm{day})}\times \;\mathrm{Vol}.\;(\mathrm{mL})$$

*Vol.*: volume of daily drink to prepare.

### 4.4. Systolic Blood Pressure Determination Procedure (SBP)

Measured at baseline and before sacrifice by tail-cuff plethysmography using a photoelectric sphygmomanometer (Grass D.C. 7DAC) and oscilloscope (Grass D.C. 79D, Grass Instruments Co., Quincy, MA, USA).

SBP was determined at baseline, prior to the initiation of any dietary or pharmacological intervention, and the animals were transferred to the experimental facility 5 days later to ensure that all animals were normotensive at the beginning of the study. During this 5-day acclimatization period, the animals were habituated to the tail-cuff procedure, including tail compression and the associated physical restraint, in order to minimize stress during blood pressure measurements. Animal handling was consistently performed by the same investigator throughout the study, allowing the animals to become habituated to the investigator’s voice, scent, and touch and thereby reducing handling-related stress and improving measurement consistency. The same habituation procedure was applied before sacrifice. SBP measurements were performed in a quiet, temperature-controlled room. The equipment was calibrated before each measurement session. For each animal, repeated measurements were obtained until five technically valid readings were recorded. A measurement was considered valid when the animal remained still during tail-cuff compression and a clear pulse signal was detected following cuff release, ensuring reliable determination of the SBP value. The SBP for each animal was calculated as the arithmetic mean of the five valid measurements.

### 4.5. Urine and Blood Collection

Three days before sacrifice, the animals were placed in metabolic cages for 48 h. During the last 24 h, urine samples were collected to perform the corresponding determinations. A urine fraction (0.5 mL) from the first 6 h collection was collected into 100 μL of 6 N HCl in order to prevent catecholamine degradation and then separated into Eppendorf tubes containing 1 μL of 1 N HCl and stored at −80 °C for L-dopa and dopamine determination by HPLC. Remaining urine during this period was added to the subsequent 18 h urine collection fraction and stored at −20 °C for sodium, creatinine, and albumin determinations (Spectrum CCX, Abbott Diagnostics, Lake Forest, IL, USA). Both collected volumes were considered to calculate 24 h diuresis.

On the day of sacrifice, following a 6 h fasting period, blood was collected directly into previously heparinized Eppendorf tubes by retro-orbital puncture under anesthesia (*ketamine* 80 mg/kg + *xylazine* 12 mg/kg; PRO-SER S.A, Capitán Sarmiento, Buenos Aires, Argentina). Then, the samples were centrifuged at 4 °C and 2700 rpm for 20 min to separate the plasma, which was then aliquoted into new, appropriately labeled Eppendorf tubes according to the volume required for the different determinations to be performed.

### 4.6. Euthanasia and Tissue Processing

After blood sample collection, while the animals were still under anesthesia, they were euthanized in a CO_2_ chamber. Following unconsciousness and absence of spontaneous movements, death was confirmed by verifying the complete absence of respiratory movements and cardiac activity before any further intervention, including tissue collection.

After euthanasia, the kidneys were removed, decapsulated, weighed, and randomly allocated for histology, transmission electron microscopy (TEM), and Western blot.

### 4.7. Measurements and Determinations

All determinations were performed by biochemists who were entirely independent of the research group. Histological and image analyses, however, were performed by the first author.

#### 4.7.1. Plasma

Glycemia: Accu-Chek Performa Nano (Roche Diagnostics GmbH, Mannheim, Germany). Triglycerides: TG Color GPO/PAP AA kit (Wiener Lab., C.A.B.A., Buenos Aires, Argentina). Insulin: ELISA (Mercodia Rat Insulin ELISA, Uppsala, Sweden). TyG [50] and HOMA-IR [53] were calculated.

#### 4.7.2. Renal Function

Sodium and creatinine in plasma and urine (Spectrum CCX); renal function was evaluated by measuring creatinine clearance (ClCr) based on the 24 h urine collection and plasma values; tubular function by urinary sodium excretion (UNa^+^.V) and fractional sodium excretion (FENa^+^).

#### 4.7.3. Urinary Catecholamines

L-Dopa and dopamine were measured by reverse-phase HPLC (Zorbax RxC18 column; DuPont, Wilmington, DE, USA; amperometric detection, ESA Biosciences, Inc., Chelmsford, MA, USA). Internal standard: 3,4-dihydroxybenzylamine (Sigma-Aldrich, Cat. No. 858781, Burlington, MA, USA). External standards: L-Dopa (Cat. No. 13248) and dopamine (Cat. No. 21992), Cayman Chemical, Ann Arbor, MI, USA. Limit of detection: 20 pg/sample. HPLC procedure was performed as follows:

(a) Intra- and inter-assay variability: Reproducibility of the assay method for L-dopa and dopamine was rigorously evaluated by repeatedly injecting extracts prepared from aliquots of the same urine sample. The intra-day coefficient of variation (CV) was 7.8% for L-dopa and 6.5% for dopamine. The inter-day CV was 10.2% and 11.5% for L-dopa and dopamine, respectively.
(b) Recovery: To account for potential sample loss during preparation, recovery was evaluated by duplicate injections of extracts obtained from catecholamine-free urine spiked with a series of various concentrations of standards of L-dopa and dopamine, along with a constant concentration of the internal standard, 3,4-dihydroxybenzylamine (DHBA).
(c) Linearity: Calibration of the instrument was carried out by injecting 20 µL of diluted extract of catecholamine-free urine spiked with 1 µg of L-dopa, 1 µg of dopamine, and 3 pg of DHBA. Linearity was established across a range of 2.5–1000 ng/mL, showing a correlation coefficient (r^2^) of 0.999 for both L-dopa and dopamine.
(d) Limit of detection (LOD): The LOD was 2.5 ng/mL for both L-dopa and dopamine.
(e) System stabilization: The use of DHBA as an internal standard effectively minimized analytical variability due to transient changes in detector sensitivity or HPLC column performance, as both the internal standard and the analytes exhibited parallel responses at the same time.

#### 4.7.4. Albuminuria

Cobas (ALBT2 Tina-quant Albumin Gen.2; Roche Diagnostics, Rotkreuz, Switzerland) kit analysis was employed to calculate the UACR [54].

#### 4.7.5. Renal Protein Expression by Western Blot

Renal cortex protein expression of LAT2, OCT2, OCTN1/2/3, D1DR, D2DR, AT1R, Na^+^/K^+^-ATPase, TGF-β1, and NF-κB1, was determined. As a loading control, β-tubulin was used. Table 8 shows the detailed data of each of the antibodies used. Samples were distributed across two independent gels according to the experimental design and the number of experimental groups analyzed. The gels were run in parallel under identical electrophoresis conditions. The transfer conditions from the gel to the corresponding nitrocellulose membrane were the same for each gel. The exposure of the membranes to the final films was also processed simultaneously using the same immunodetection and chemiluminescent exposure conditions: In a darkroom equipped with an appropriate red safelight, the membranes were treated with a mixture of distilled water, p-coumaric acid (Sigma-Aldrich, Cat. No. C9008, USA), luminol (Sigma-Aldrich, Cat. No. 123072, USA), hydrogen peroxide, and Tris buffer (pH 8.8). The membranes were then exposed to X-ray film (Carestream Health Inc. T-MAT G/RA, Cat. No. 186 9080, Rochester, NY, USA) for 1 to 5 min, depending on the chemiluminescent signal intensity of each membrane, using an Amersham™ Hypercassette™ autoradiography cassette (Cytiva, Marlborough, MA, USA). Finally, the films were developed using Romek TD-3 developer (1:10 dilution) and fixed with Romek TD-4 fixer (1:10 dilution). Densitometric analysis was performed using ImageJ 1.54p software. An identical region of interest (ROI) was established at the beginning of the analysis and applied consistently to all corresponding bands. For each animal, protein abundance was expressed as the ratio of the target-protein band intensity to the corresponding β-tubulin band intensity from the same lane (target/β-tubulin), and these normalized values were used for statistical analysis. Since several of the target proteins of the primary antibodies exhibited similar molecular weights, membranes were stripped twice with 5% acetic acid after primary antibody detection to probe for the loading control (to achieve this, the membrane of interest was incubated twice for 30 min with 5% acetic acid in distilled water, followed by 5 washes with 1× TBS—0.1% Tween buffer). Probing proteins of identical molecular weight on the same membrane requires complete removal of the initial antibody signal to avoid signal compounding. To prevent quantification errors, we strictly implemented a stripping verification step in our laboratory workflow. After the acetic acid treatment and prior to incubation with the anti-β-tubulin antibody, membranes were incubated exclusively with the secondary HRP-conjugated antibody and exposed to the chemiluminescence substrate. In all cases, no residual bands or signals were detected at 50 kDa, validating that the primary target antibody had been completely stripped and that the subsequent signal belonged solely to β -tubulin. The β-tubulin bands were clearly detectable on the original X-ray films following manual development.

#### 4.7.6. Renal Conventional Histology and Ultrastructure

Cortical fragments processed and examined for renal histology and TEM were evaluated qualitatively and descriptively for cellular and ultrastructural alterations.

##### Light Microscopy

Sections (8 µm thick) were stained with hematoxylin–eosin for routine histological assessment and with Picrosirius Red to visualize interstitial collagen deposition [55]. Digital images of Picrosirius Red-stained sections were acquired using a Nikon Type 104c microscope equipped with a digital camera under conventional bright-field illumination. Cortical fibrosis was quantified in 20 non-overlapping fields per animal using ImageJ and expressed as the percentage of Picrosirius Red-positive interstitial area relative to the total cortical area, after exclusion of vascular areas. Similar bright-field image-based approaches have been used for the quantitative assessment of renal interstitial collagen deposition in experimental models using Picrosirius Red staining and digital image analysis [55,56,57].

*Cortical fibrosis* (%) = [*Σ cortical interstitial collagen*/(*total area* − *Σ vessels*)] × 100

##### Transmission Electron Microscopy

Sections of 1 mm^3^ of cortical fragments were processed for TEM and examined with a Zeiss EM 109T equipped with a Gatan ES1000W digital camera.

### 4.8. Statistical Analysis

Statistical analyses were performed using InfoStat software version 2014. For comparisons involving two groups, Student’s *t*-test was used, and two-way ANOVA was used to assess the influence of two different categorical independent variables (diet and treatment) on each continuous dependent variable, followed by Tukey’s multiple-comparisons test. The comparisons of primary biological interest were C vs. HFD, HFDL vs. HFD, and CL vs. C. Pearson’s correlation analyses and linear regression analyses were performed to assess relationships between the indicated variables. Data are presented as Mean ± SD, and statistical significance was set at *p* < 0.05. Graphs were generated using GraphPad Prism v11.1.0 (218).

## Figures and Tables

**Figure 1:**
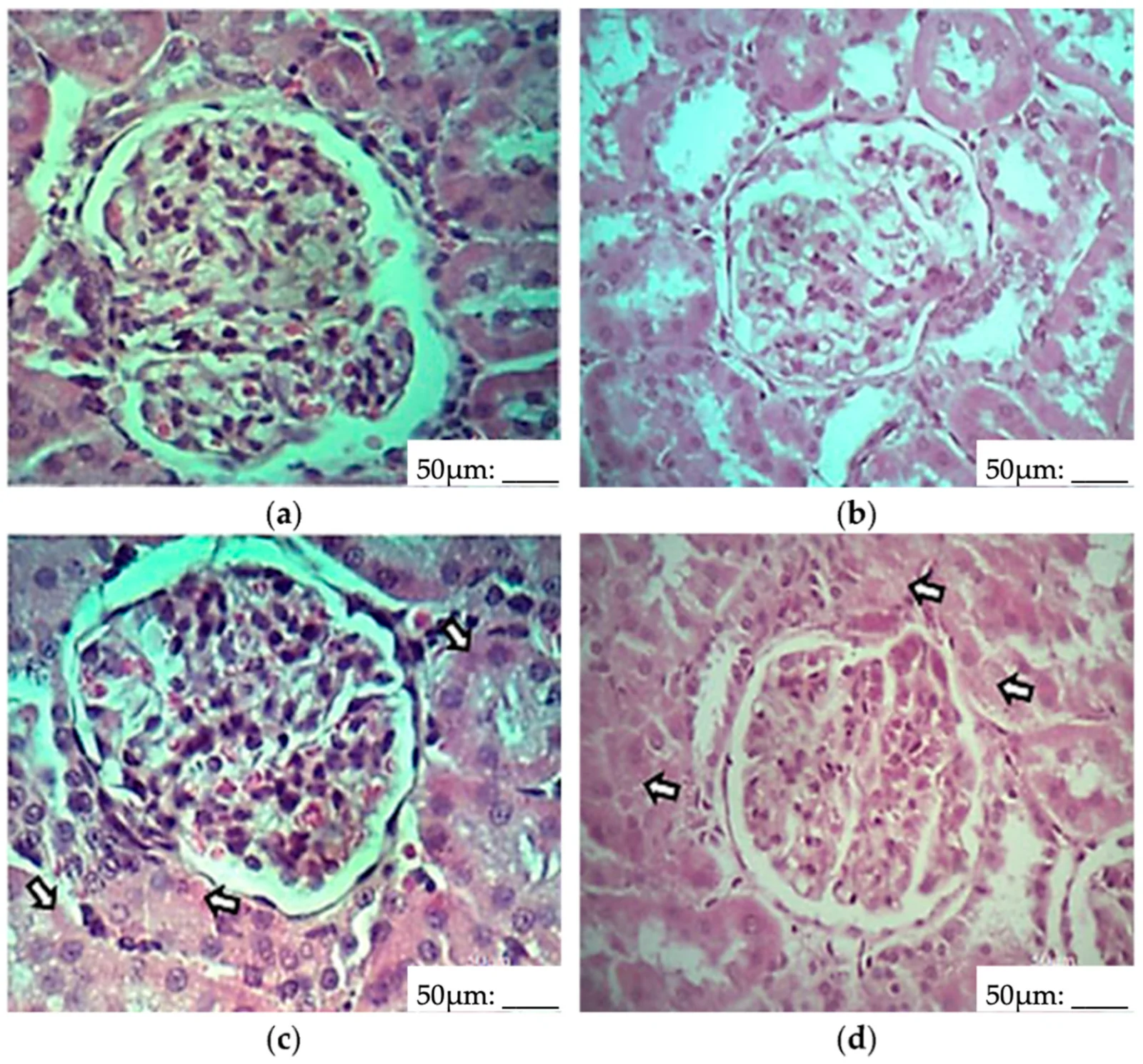
Light microscopy, hematoxylin–eosin stained, total magnification 400×. (**a**) C; (**b**) CL; (**c**) HFD; (**d**) HFDL. White arrows in (**c**,**d**) indicate tubular cell cytoplasmic vacuoles.

**Figure 2:**
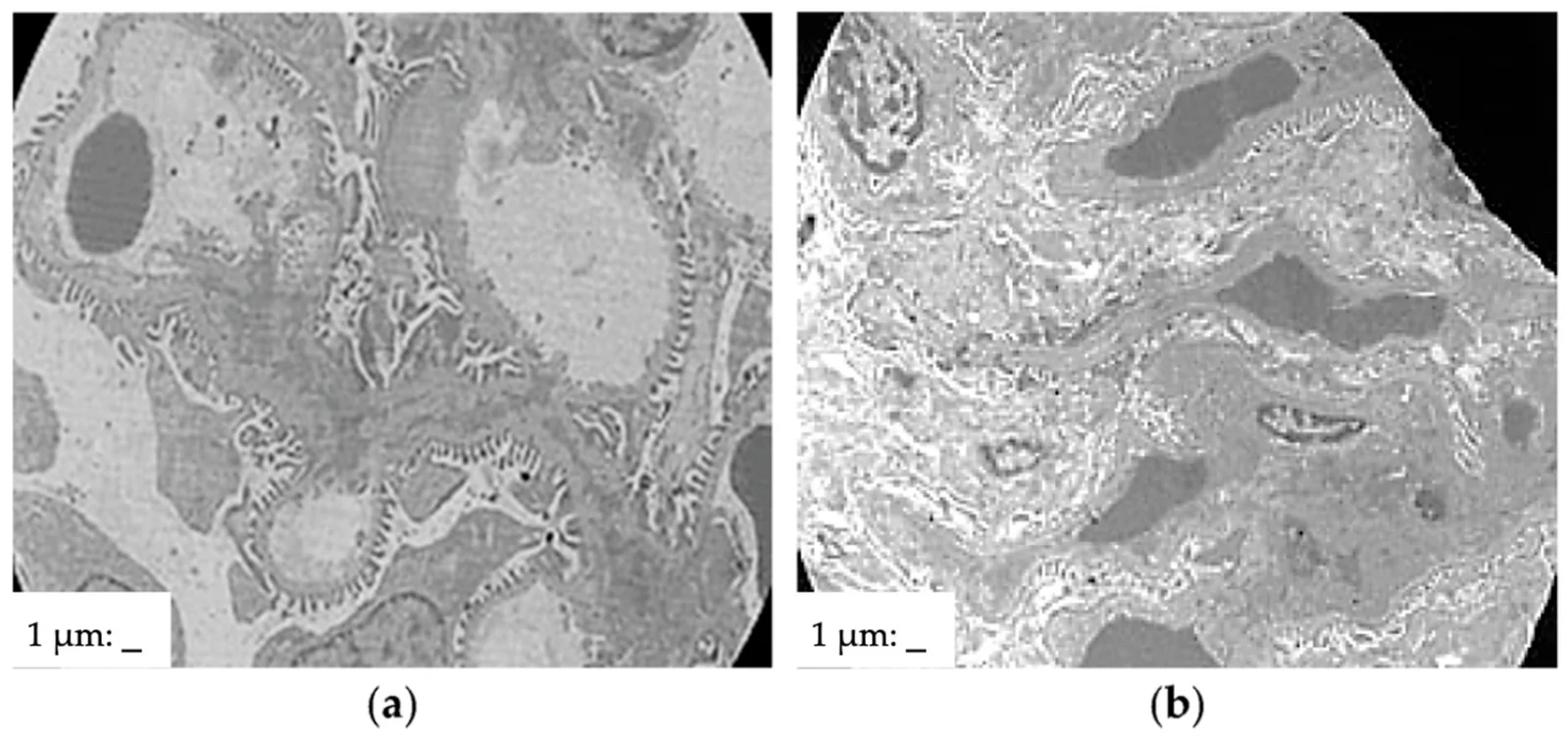

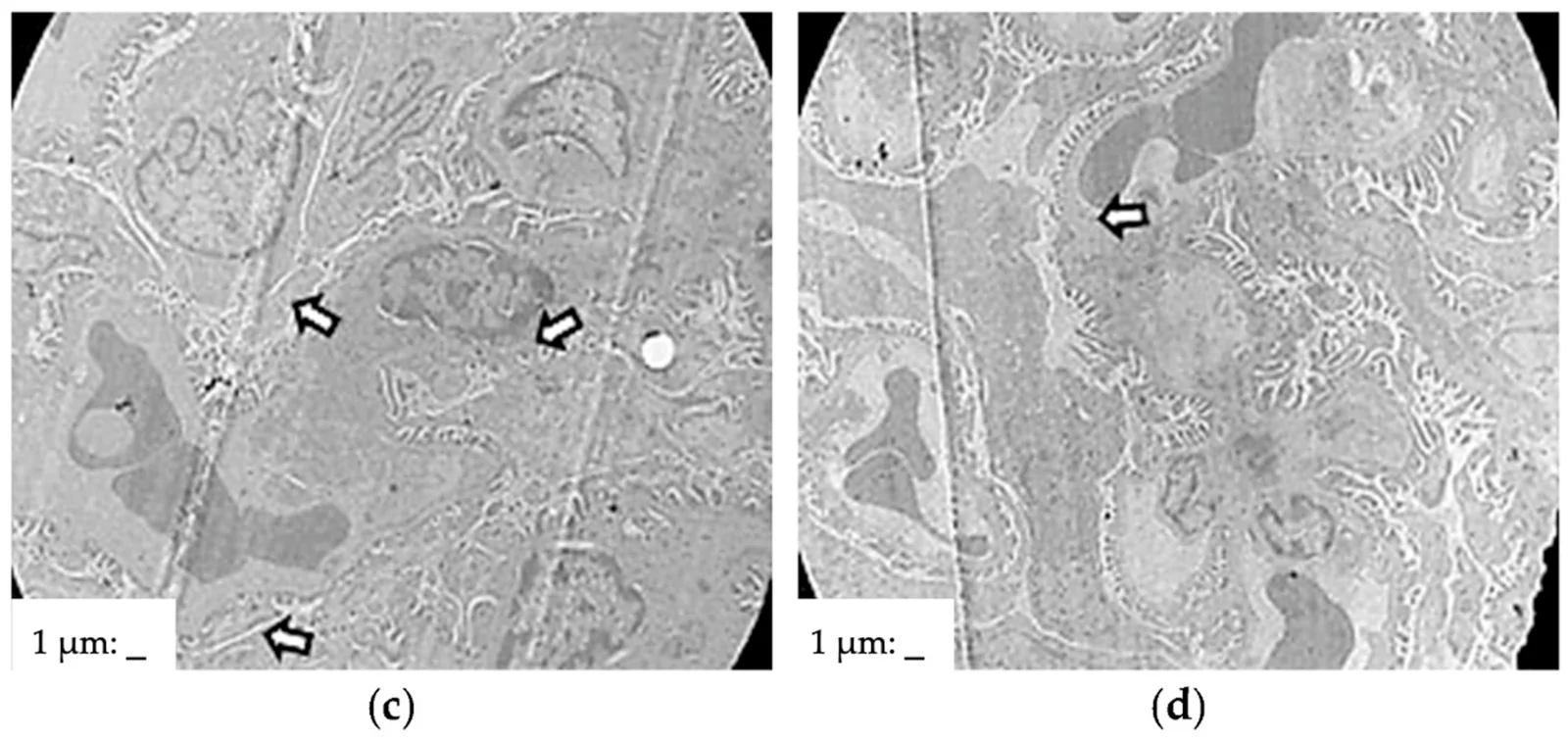
Representative TEM images showing glomerular ultrastructural features in the experimental groups. The images illustrate the general morphology of the glomerular filtration barrier and podocyte foot processes, including the apparent foot process effacement observed in HFD animals. The micrographs are presented as representative qualitative observations and were not subjected to quantitative morphometric analysis. Total magnification: 7000×. (**a**) C; (**b**) CL; (**c**) HFD; (**d**) HFDL. White arrows in (**c**,**d**) indicate podocyte foot processes with altered morphology.

**Figure 3:**
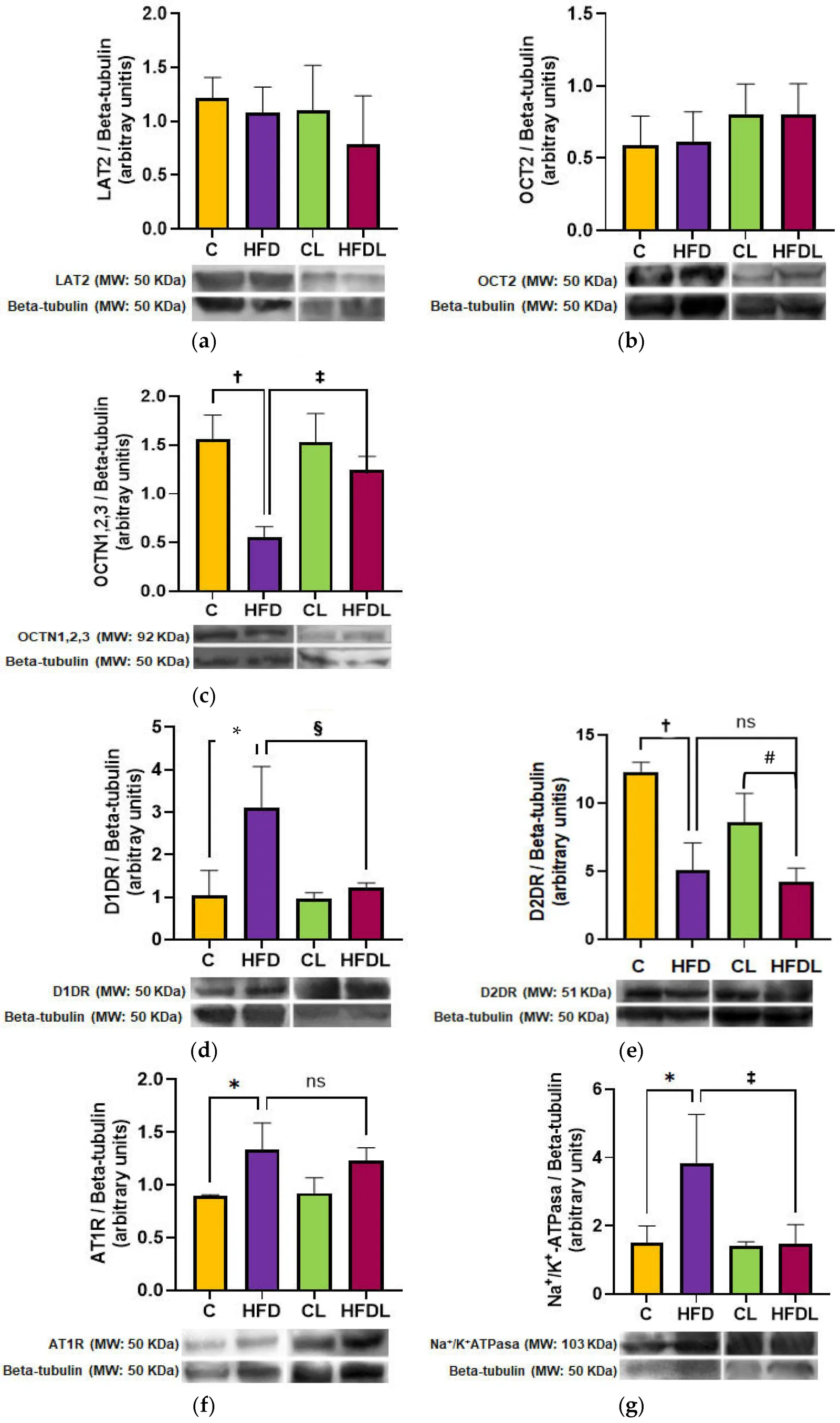
Western blot analysis of RDS and RAS proteins and Na^+^/K^+^-ATPase in renal cortex. (**a**) LAT2; (**b**) OCT2; (**c**) OCTN1,2,3; (**d**) D1DR; (**e**) D2DR; (**f**): AT1R; (**g**) Na^+^/K^+^-ATPase. Data: Mean ± SD. Significance: * *p* < 0.05 C vs. HFD; ^†^ *p* < 0.01 C vs. HFD; ^‡^ *p* < 0.05 HFDL vs. HFD; ^§^ *p* < 0.01 HFDL vs. HFD; ^#^ *p* < 0.05 CL vs. HFDL; ns: non significant. Due to the experimental design, C and HFD samples and CL and HFDL samples were analyzed on two independent membranes processed in parallel under identical experimental conditions. The vertical dividing line indicates the boundary between the two independent membranes. Horizontal dividing lines indicate physical membrane sectioning performed before antibody incubation, allowing detection of proteins with different molecular weights.

**Figure 4:**
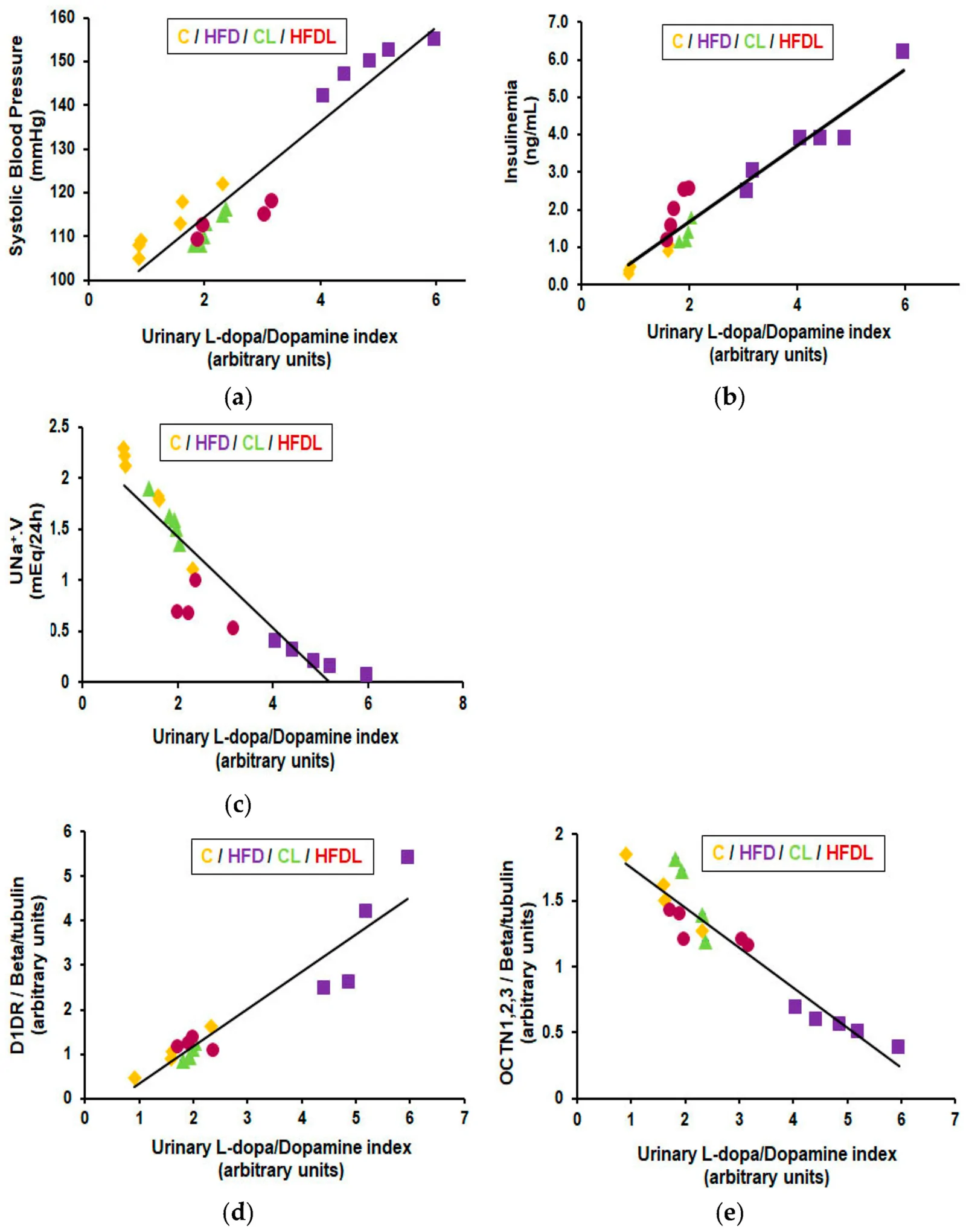
Functional correlations with the L-Dopa/dopamine index. (**a**) SBP vs. L-Dopa/dopamine index, r = 0.95, R^2^ = 0.90, *p* < 0.01; (**b**) insulinemia vs. L-Dopa/dopamine index, r = 0.96, R^2^ = 0.91, *p* < 0.01; (**c**) UNa^+^.V vs. L-Dopa/dopamine index, r = 0.91, R^2^ = 0.83, *p* < 0.01; (**d**) OCTN1,2,3 vs. L-Dopa/dopamine index, r = 0.95, R^2^ = 0.91,  *p* < 0.01; (**e**) D1DR vs. L-Dopa/dopamine index, r = 0.95, R^2^ = 0.89, *p* < 0.05.

**Figure 5:**
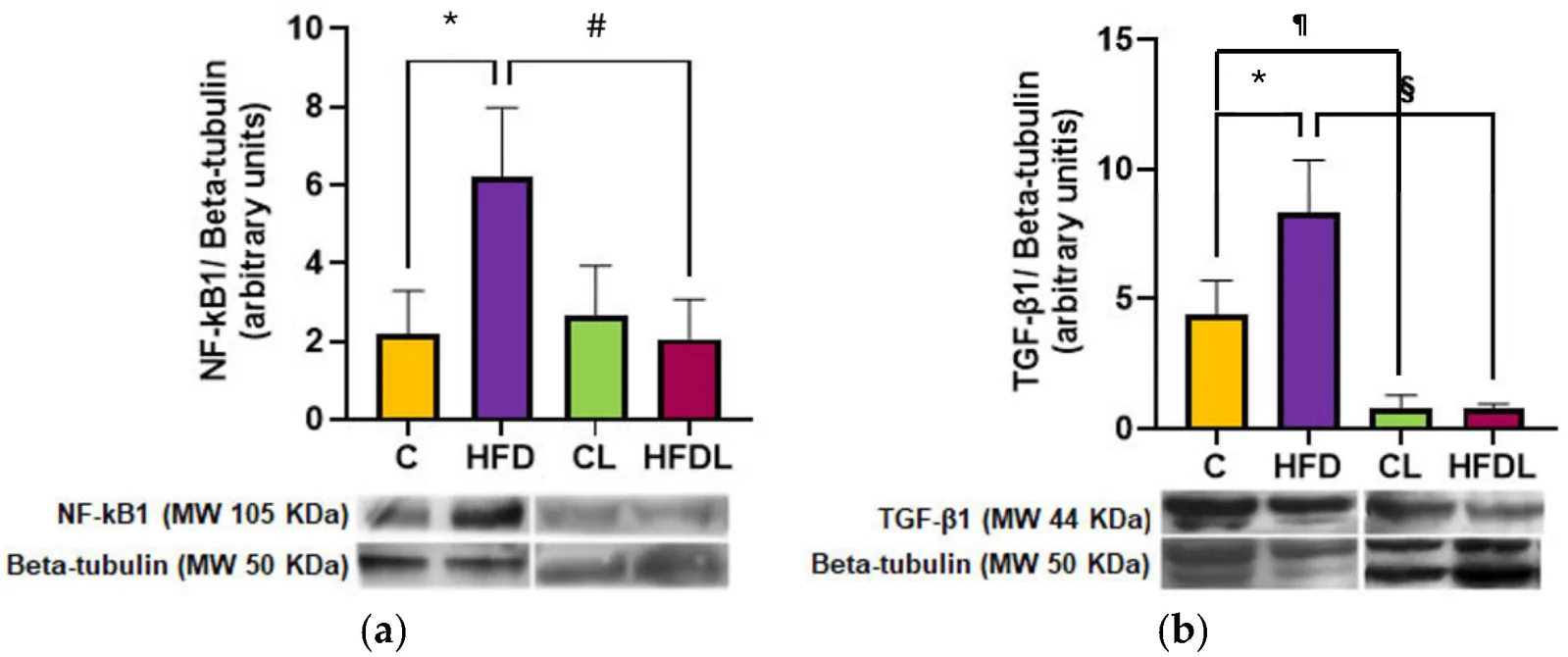
Inflammatory markers (Western blot). (**a**) NF-κB1; (**b**) TGF-β1. Data: Mean ± SD. Significance: * *p* < 0.05 C vs. HFD; ^§^ *p* < 0.01 HFDL vs. HFD; ^#^ *p* < 0.05 CL vs. HFDL; ^¶^ *p* < 0.01 CL vs. C. Due to the experimental design, C and HFD samples and CL and HFDL samples were analyzed on two independent membranes processed in parallel under identical experimental conditions. The vertical dividing line indicates the boundary between the two independent membranes. Horizontal dividing lines indicate physical membrane sectioning performed before antibody incubation, allowing detection of proteins with different molecular weights.

**Figure 6:**
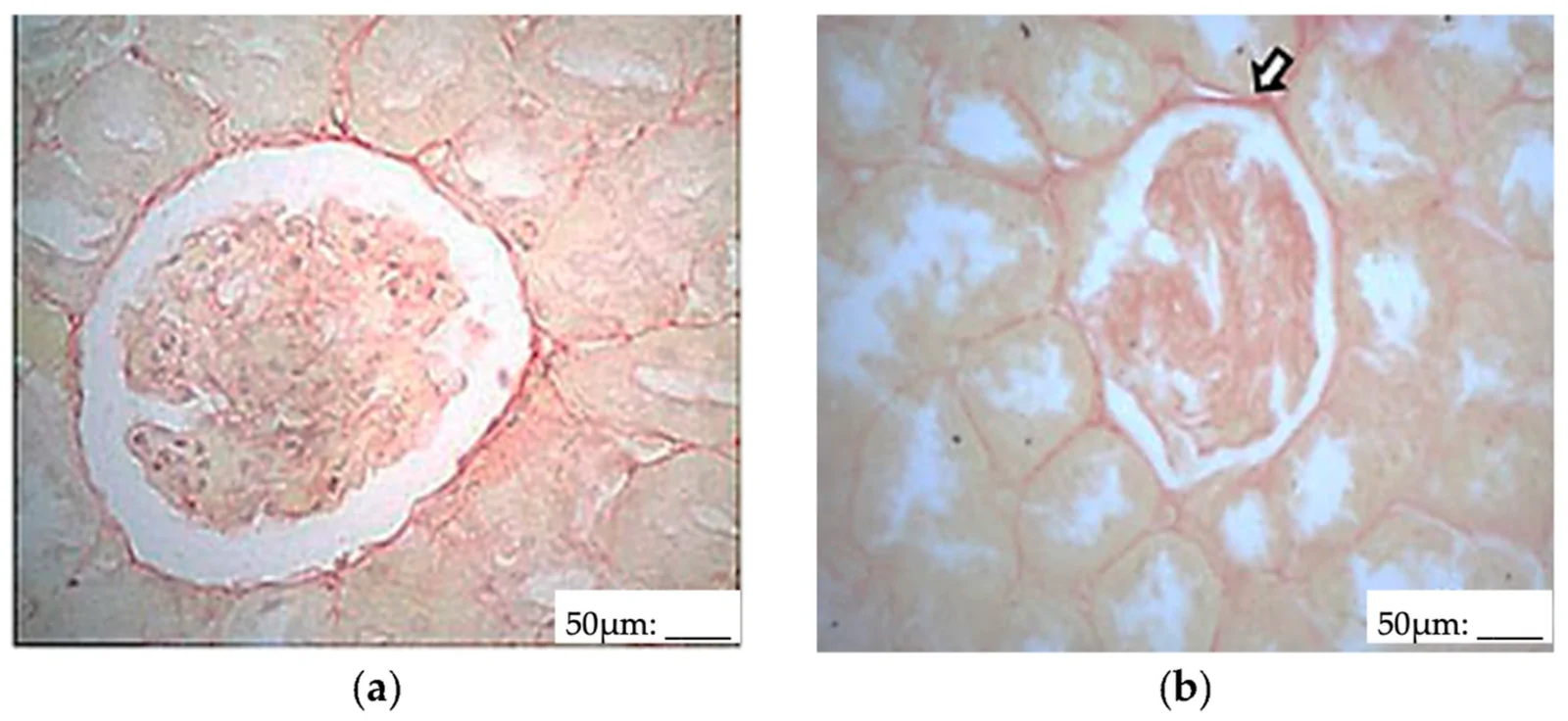

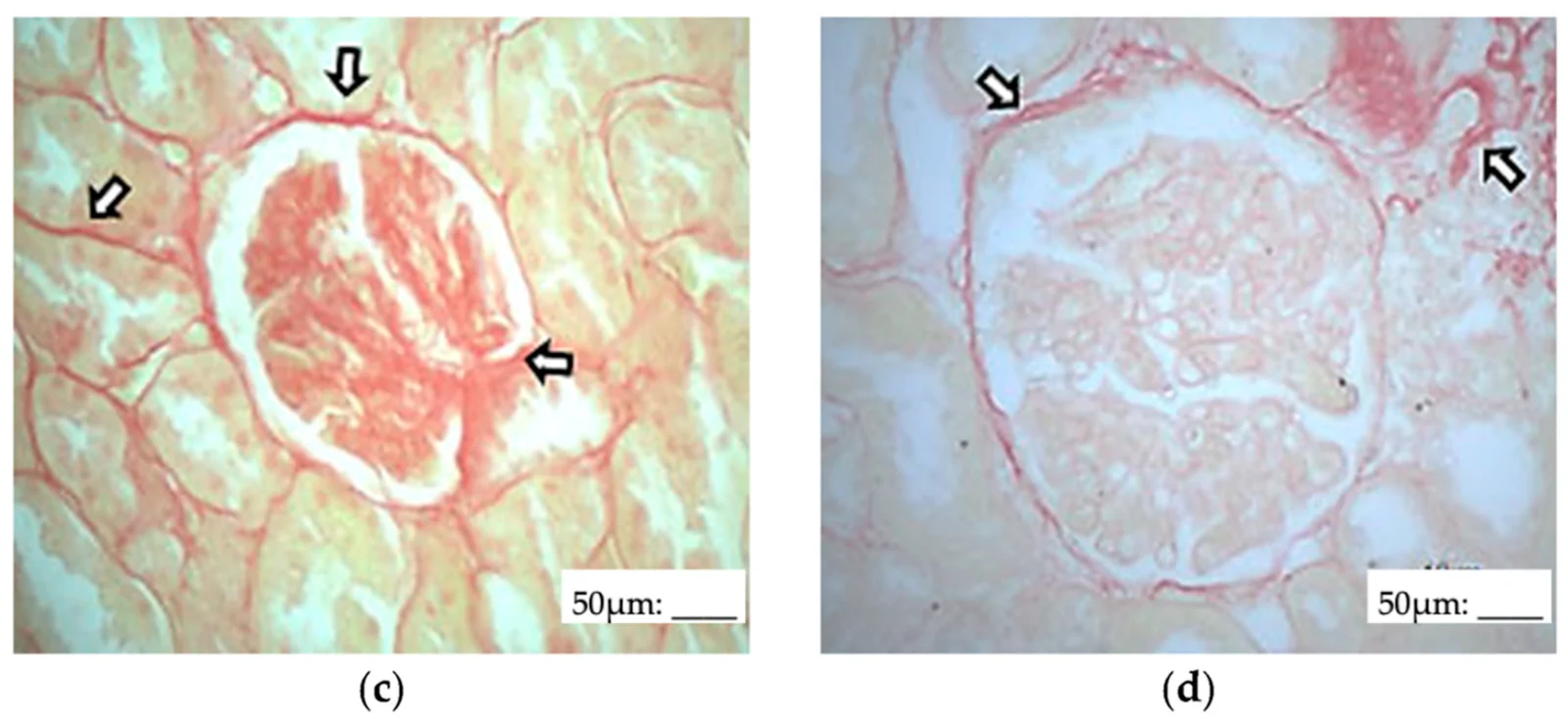
Interstitial fibrosis (picrosirius red) in renal cortex. (**a**) C; (**b**) CL; (**c**) HFD; (**d**) HFDL. White arrows indicate sites of increased interstitial fibrosis. Total magnification: 400×.

**Figure 7:**
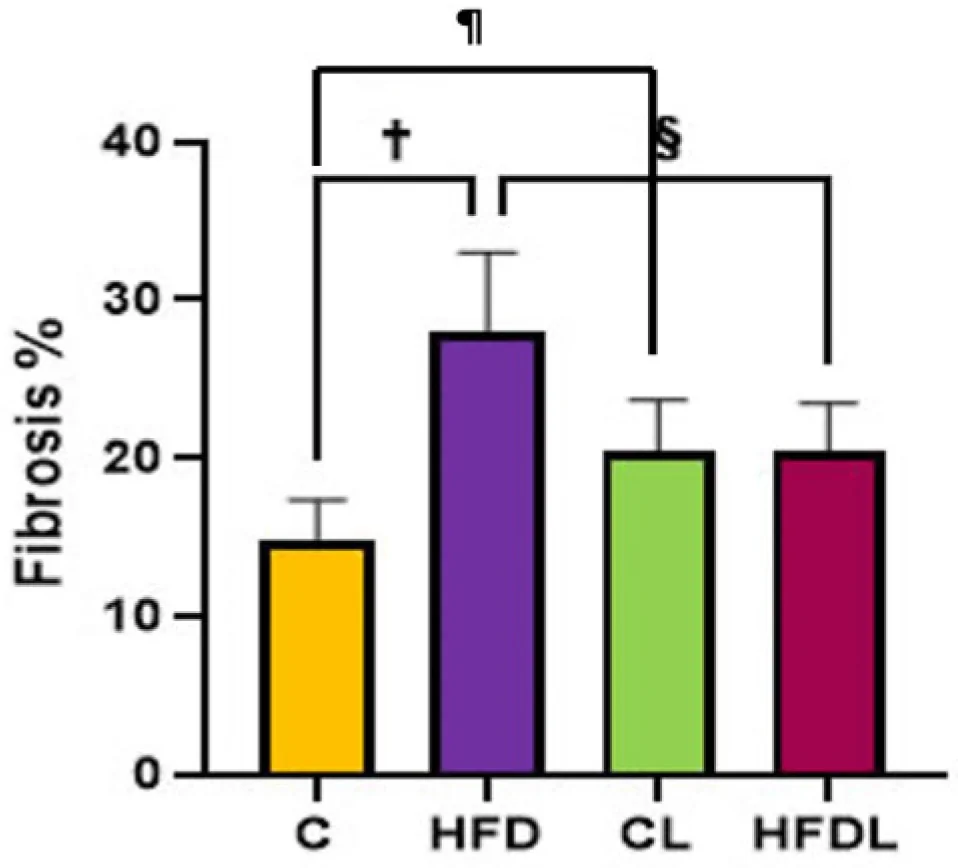
Fibrosis % in renal cortex. Data: Mean ± SD. Significance: ^†^ *p* < 0.01 C vs. HFD; ^§^ *p* < 0.01 HFDL vs. HFD; ^¶^ *p* < 0.01 CL vs. C.

**Table 1:** Body weight, food/fluid/calorie intake, and plasma metabolic parameters.

Parameter	C (n = 4)	HFD (n = 6)	CL (n = 4)	HFDL (n = 6)
Body weight (g)	415.7 ± 48.9	473.8 ± 39.1 *	389.7 ± 40.5	359.1 ± 38.2 ^‡^
Food intake (g/24 h)	20.9 ± 10.1	13.8 ± 2.2 ^†^	28.4 ± 9.7	18.6 ± 4.8 ^‡^
Water intake (mL/24 h)	12.5 ± 2.6	12.4 ± 4.8	20.5 ± 4.5	19.1 ± 1.7
Caloric intake (kcal/24 h)	60.5 ± 26.9	103.6 ± 28.4 *	72.4 ± 5.9	119.3 ± 10.5 ^‡^
Triglycerides (mg/dL)	59.7 ± 15.3	119.3 ± 9.6 ^†^	53.2 ± 10.2 ^#^	83.0 ± 13.9 ^§^
Glucose (mg/dL)	117.5 ± 17.3	152.2 ± 12.3 *	100.3 ± 23.0 ^&^	173.7 ± 16.6 ^‡^
TyG index (arbitrary units)	8.0 ± 0.4	10.5 ± 0.1 ^†^	8.1 ± 0.5	10.2 ± 0.5 ^‡^
Insulin (ng/mL)	1.2 ± 0.5	4.2 ± 0.5 ^†^	1.3 ± 0.3	2.8 ± 0.1 ^‡ £^
HOMA-IR (arbitrary units)	0.7 ± 0.1	3.3 ± 0.6 ^†^	1.5 ± 0.7	3.2 ± 0.5

Data: Mean ± SD. Significance: * *p* < 0.05 C vs. HFD; ^†^ *p* < 0.01 C vs. HFD; ^‡^ *p* < 0.05 HFDL vs. HFD; ^§^ *p* < 0.01 HFDL vs. HFD; ^&^ *p* < 0.05 CL vs. C; ^#^ *p* < 0.05 CL vs. HFDL; ^£^ *p* < 0.01 HFDL vs. C.

**Table 2:** Systolic blood pressure, urinary L-Dopa/dopamine index, and renal function parameters.

Parameter	C (n = 4)	HFD (n = 6)	CL (n = 4)	HFDL (n = 6)
SBP (mmHg)	120.7 ± 1.4	134.8 ± 7.6 ^†^	106.4 ± 6.7 ^¶^	109.5 ± 5.8 ^§^
Urinary L-Dopa/dopamine index (arbitrary units)	1.1 ± 0.4	2.9 ± 0.8 ^†^	1.8 ± 0.3	1.8 ± 0.2 ^‡^
Urinary albumin/creatinine ratio (mg/g)	17.4 ± 2.3	12.9 ± 1.2	15.3 ± 1.7	8.5 ± 1.7
Diuresis (mL/24 h)	16.4 ± 3.6	3.5 ± 0.7 ^†^	17.3 ± 3.3	6.23 ± 2.5 ^‡^
ClCr (mL/min)	1.7 ± 0.4	1.4 ± 0.4	1.8 ± 0.3	1.4 ± 0.2
FENa^+^ (%)	0.43 ± 0.06	0.23 ± 0.01 ^†^	0.51 ± 0.04	0.39 ± 0.06 ^‡^
UNa^+^.V (mEq/24 h)	1.8 ± 0.3	0.5 ± 0.2 ^†^	2.0 ± 0.3	0.8 ± 0.1 ^‡^

Data: Mean ± SD. Significance: ^†^ *p* < 0.01 C vs. HFD; ^‡^ *p* < 0.05 HFDL vs. HFD; ^§^ *p* < 0.01 HFDL vs. HFD; ^¶^ *p* < 0.01 CL vs. C.

**Table 3:** Western blot optical densitometric analysis results for RDS and RAS proteins and Na^+^/K^+^-ATPase in renal cortex.

Protein (Arbitrary Units)	C (n = 4)	HFD (n = 6)	CL (n = 4)	HFDL (n = 6)
LAT2	1.22 ± 0.19	1.08 ± 0.24	1.10 ± 0.42	0.78 ± 0.45
OCT2	0.59 ± 0.25	0.61 ± 0.21	0.80 ± 0.21	0.80 ± 0.22
OCTN1,2,3	1.56 ± 0.24	0.55 ± 0.11 ^†^	1.53 ± 0.29	1.82 ± 0.14 ^‡^
D1DR	1.04 ± 0.58	3.12 ± 0.96 *	0.96 ± 0.14	1.21 ± 0.12 ^§^
D2DR	12.32 ± 0.70	5.13 ± 1.8 ^†^	8.60 ± 2.14	4.27 ± 0.99 ^#^
AT1R	0.89 ± 0.01	1.34 ± 0.25 *	0.92 ± 0.14	1.23 ± 0.13
Na^+^/K^+^-ATPase	1.50 ± 0.50	3.83 ± 1.40 *	1.41 ± 0.13	1.47 ± 0.56 ^‡^

Data: Mean ± SD. Significance: * *p* < 0.05 C vs. HFD; ^†^ *p* < 0.01 C vs. HFD; ^‡^ *p* < 0.05 HFDL vs. HFD; ^§^ *p* < 0.01 HFDL vs. HFD; ^#^ *p* < 0.05 CL vs. HFDL.

**Table 4:** Western blot optical densitometric analysis results for inflammatory proteins.

Protein (Arbitrary Units)	C (n = 4)	HFD (n = 6)	CL (n = 4)	HFDL (n = 6)
NF-κB1	2.20 ± 1.08	6.21 ± 1.77 *	2.68 ± 1.26	2.05 ± 1.02 ^‡^
TGF-β1	4.43 ± 1.29	8.32 ± 2.04 *	0.82 ± 0.47 ^¶^	0.78 ± 0.19 ^§^

Data: Mean ± SD. Significance: * *p* < 0.05 C vs. HFD; ^‡^ *p* < 0.05 HFDL vs. HFD; ^§^ *p* < 0.01 HFDL vs. HFD; ^¶^ *p* < 0.01 CL vs. C.

**Table 5:** Standard rodent diet composition.

Nutrient	Quantity/100 g (%)
Starch	69%
Protein	20%
Minerals	6%
Fat	3%
Fiber	2%
Vitamins supplements	Necessary amounts
Calories	3.3 kcal/g
Food Supplier: ACA Nutrición Animal. Asociación de Cooperativas Argentinas C.L., San Nicolás, Provincia de Buenos Aires, Argentina.	

**Table 6:** Bovine fat composition.

Nutrient	Quantity/100 g (%)
Total fat	99%
Saturated	77%
Trans	19%
Carbohydrates, protein, fiber, sodium, vitamins and minerals supplements	Necessary amounts
Calories	9.0 kcal/g
Food Supplier: Faty, Quickfood S.A., San Jorge, Provincia de Santa Fe, Argentina.	

**Table 7:** Detailed losartan information.

Characteristic	Detail
Losartan chemical form	Potassium losartan
Purity	100% dry basis
Drug Supplier: Droguería Saporiti S.A.C.I.F.I.A., C.A.B.A., Buenos Aires, Argentina	

**Table 8:** Primary antibodies used for Western blot.

Protein	Antibody	Dilution	Molecular Weight (kDa)	Supplier and Catalog N°
D1DR	Rabbit polyclonal anti-D1DR	1:1000	50	Proteintech Group, Inc., Rosemont, IL, USA 17934-1-AP
D2DR	Rabbit polyclonal anti-D2DR	1:600	51	Proteintech Group, Inc., Rosemont, IL, USA 55084-1-AP
OCTN1,2,3	Rabbit polyclonal anti-OCTN 1,2,3	1:2000	90	Santa Cruz Biotechnology, Dallas, TX, USA sc-33534
OCT2	Goat polyclonal anti-OCT2	1:800	50	Santa Cruz Biotechnology, Dallas, TX, USA sc-19814
LAT2	Goat polyclonal anti-LAT2	1:800	50	Santa Cruz Biotechnology, Dallas, TX, USA sc-27581
AT1R	Rabbit polyclonal anti-AT1R	1:1000	50	Proteintech Group, Inc., Rosemont, IL, USA 25343-1-AP
Na^+^/K^+^-ATPase (α1)	Rabbit polyclonal anti-α1 Na^+^/K^+^-ATPase	1:10,000	105	Abcam, Cambridge, UK ab74945
NF-κB1	Rabbit polyclonal anti-NF-κB1	1:1000	50 and 105	Proteintech Group, Inc., Rosemont, IL, USA 14220-1-AP
TGF-β1	Rabbit polyclonal anti-TGF-β1	1:500	44	Proteintech Group, Inc., Rosemont, IL, USA 21898-1-AP
β-tubulin	Rabbit polyclonal anti-β-tubulin	1:3000	50	Abcam, Cambridge, UK ab6046

## Data Availability

Data presented in the article can be made available on request.

## Abbreviations

The following abbreviations are used in this manuscript:

AT1R	Type 1 receptor for Angiotensin II
C	Control group
CL	Control plus losartan group
ClCr	Creatinine Clearance
D1DR	Dopamine type 1 receptors
D2DR	Dopamine type 2 receptors
FENa^+^	Sodium fractional excretion
HFD	High fat diet group
HFDL	High fat diet plus losartan group
HOMA-IR	Homeostatic Model Assessment for Insulin Resistance
L	Losartan
LAT	L-amino acid transporter
OCT2	Organic cation transporter type 2
OCTN1,2,3	Novel organic cation transporter
SBP	Systolic blood pressure
SFA	Saturated fatty acids
SOD	Superoxide dismutase
TyG	Triglyceride–Glucose index
UNa^+^.V	Daily urinary sodium excretion
UACR	Urinary Albumine to Creatinine Ratio
